# Computer simulations of the mechanical response of brushes on the surface of cancerous epithelial cells

**DOI:** 10.1038/srep13218

**Published:** 2015-08-28

**Authors:** A. Gama Goicochea, S. J. Alas Guardado

**Affiliations:** 1 Instituto de Física, Universidad Autónoma de San Luis Potosí Álvaro Obregón 64, San Luis Potosí 78000, Mexico; 2 Departamento de Ciencias Naturales, Universidad Autónoma Metropolitana Unidad Cuajimalpa, Av. Vasco de Quiroga 4871, Distrito Federal 05300, Mexico

## Abstract

We report a model for atomic force microscopy by means of computer simulations of molecular brushes on surfaces of biological interest such as normal and cancerous cervical epithelial cells. Our model predicts that the force needed to produce a given indentation on brushes that can move on the surface of the cell (called “liquid” brushes) is the same as that required for brushes whose ends are fixed on the cell’s surface (called “solid” brushes), as long as the tip of the microscope covers the entire area of the brush. Additionally, we find that cancerous cells are softer than normal ones, in agreement with various experiments. Moreover, soft brushes are found to display larger resistance to compression than stiff ones. This phenomenon is the consequence of the larger equilibrium length of the soft brushes and the cooperative association of solvent molecules trapped within the brushes, which leads to an increase in the osmotic pressure. Our results show that a careful characterization of the brushes on epithelial cells is indispensable when determining the mechanical response of cancerous cells.

The nature of brushes that cover cells and biological membranes is important for the understanding of the differences between cancerous and normal cells, for example^1^,^2^. Several recent studies report that cancerous cells are softer than normal ones^3^,^4^,^5^,^6^, however, the mechanisms that give rise to these differences are still poorly understood. One the most popular modern techniques used for the study of cell and molecular biology is atomic force microscopy (AFM). In recent years, AFM has been used to determine cell velocity, structure and association at the sub—micron level, yielding extremely valuable information^7^,^8^,^9^,^10^. The insights provided by AFM are rich and novel because experiments can be done *in vivo*^11^,^12^,^13^, controlling variables such as temperature, solvent quality and ionic strength, to name a few. Various recent reports reveal that cancerous cells are softer than normal ones^3^,^4^,^5^,^6^,^9^,^14^, while other groups have argued that the mechanical response of different types of cancer is tissue—specific^15^,^16^. Therefore, it is of paramount importance to determine the effects of the comparative softness of cancer cells, which has far—reaching implications for the detection, diagnosis and perhaps even for the treatment and prevention of cancer, particularly in epithelial cells such as those found in the cervical cavity. The mechanical response of brushes on cancerous human cervical epithelial cells to the AFM tip was measured by Iyer and collaborators^17^, who found differences with respect to the response of the brushes on normal (healthy) cells. These differences were attributed to the presence of brushes with non-uniform length on the cancerous cells’ surface. This is important because AFM measurements can potentially pave the way for the future design of non-invasive detection methods in epithelial cancer, and this technique can be used as a supporting diagnosis tool. Also, ref. 17 is the first report in which the mechanical response of the brush layer is accounted for separately from that of the cell’s surface. Additionally, the brushes on the surfaces of cells, which can be microridges, microtubules, or microvilli, can behave as polymer brushes which have numerous applications, e.g. as colloid dispersants^18^, rheology modifiers^19^, and as stimuli-responsive agents^20^, whose characteristics are of interest to a wide audience in the soft matter, biomaterials, polymer science and nanotechnology communities.

We have performed computer simulations of “liquid” and “solid” brushes^21^, with the latter being brushes whose attached ends are fixed to the cell surface, while in the former case the ends of the brushes are free to move on a surface (*xy*-plane in Fig. 1(a,b)). The motivation for the modeling of both types of brushes stems from the fact that biological brushes, such as those on normal and cancerous cells belong to the liquid type, while solid brushes are the result of chemical adsorption and are usually found in nanotechnological applications, such as “smart” coatings^22^. To represent normal and cancerous cell surfaces, we used a soft model that allows for the deformation of the surfaces upon compression, while the brushes were modeled as chains of uniform (constant) length, as well as non—uniform length, motivated by the experiments of Iyer and collaborators^17^. The simulation model used, known as dissipative particle dynamics (DPD)^23^,^24^, is a coarse—grained method that involves the grouping of several microscopic units (atoms, molecules) into beads, which interact with each other according to simple rules. This technique has been chosen here because it has several advantages over more traditional, microscopic molecular dynamics simulations^25^. All—atom simulations are useful for the modeling of systems at the few nanometer scale, for up to a few nanoseconds. However, to model appropriately biological systems one needs to reach mesoscopic length scales, which still is prohibitively demanding with microscopic simulations. Coarse—graining is justified for the computational modeling of brushes on cells because the atomistic details of the membrane and brushes are not directly responsible for their physicochemical characteristics, such as the force measured with an AFM. The DPD particles are sections of fluid rather than atoms or molecules and obey simple, short—range forces, which is why much larger length and time scales can be reached. In fact, DPD can be five times more efficient than atomistic simulations^26^. Another advantage of DPD over other mesoscopic techniques is that the momentum—conserving nature of its forces allows for the preservation of hydrodynamic effects in the fluid^23^, which are important in applications such as the one reported in this work. DPD has been shown to be a very successful tool for the prediction of equilibrium and dynamic properties of polymer brushes and other biomembrane systems^27^,^28^.

## Results and Discussion

**Figure 1:**
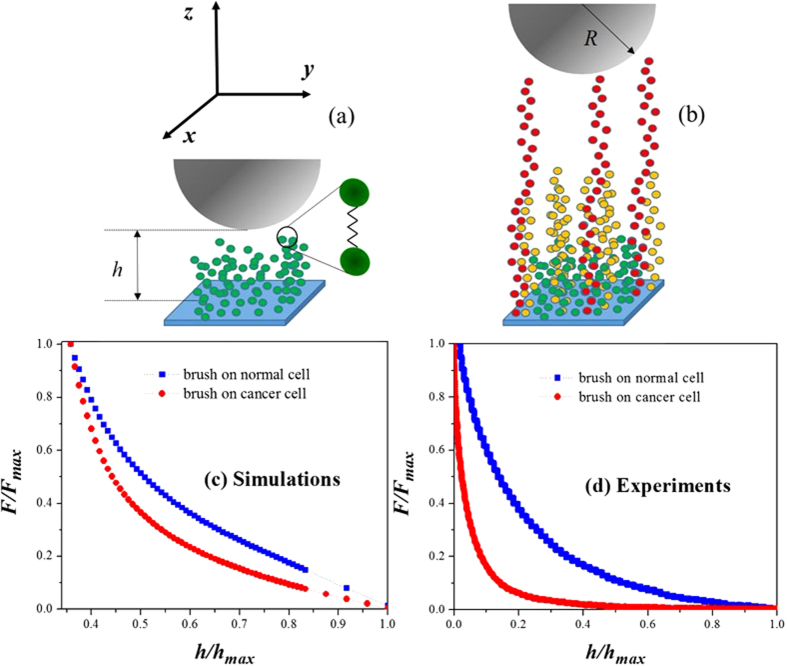
Model of brushes on normal and cancerous epithelial cells and their force profiles. (**a**) Model for normal cervical epithelial cells, covered by a uniform brush made up of chains of the same size, which are made of beads joined by springs, as illustrated by the diagram. (**b**) Model for cancerous cells whose surface is covered by brushes of three different lengths, following experimental evidence^17^. The hemisphere of radius *R* represents the AFM probe and it is not drawn to scale because the probe has a much larger area of contact with the cells surface than that shown in the figure, for clarity. (**c**) Force profiles for brushes on normal and cancerous cells predicted by our simulations. The *y*—axis represents the force (*F*) that needs to be applied to obtain a deformation *h* of the cells’ brushes. Both axes are normalized by their maximum values. The number of beads that make up the chains (*N*), as well as the number of chains per unit area on the normal cell surface (Γ) are *N* = 27, and Γ = 0.78 nm^−2^; for all brushes on cancerous cells *N*_1_ = 5, *N*_2_ = 30, *N*_3_ = 42 and Γ_1_ = 1.76 nm^−2^, Γ_2_ = 0.49 nm^−2^, Γ_3_ = 0.20 nm^−2^. (**d**) Force profiles obtained from AFM experiments on human cervical epithelial normal and cancerous cells^17^. Both axes are normalized by their maximum values.

The surfaces of cancerous epithelial cells are known to be different from the surfaces of normal cells^29^. For example, as the disease progresses (cervical intraepithelial neoplasia) there appears an increase in the nuclear—cytoplasmic ratio and the layer of flattened cells is reduced^30^. Therefore, one should in principle model differently the surfaces of healthy and cancerous cells, but we have used the same soft—surface model for both types of cells because the focus of our work is on the properties of the brushes that cover those cells. The brushes we have studied consist of a number of linear chains, made up of beads joined by freely rotating harmonic springs, attached (“grafted”) from one of their ends to the cell’s surface, see Fig. 1(a,b). Stiff brushes are constructed identically to soft brushes, with the only difference being that the spring constant (*κ*) joining the beads that make up the brush molecules of the so called “stiff” brushes is twenty times larger than the spring constant of the “soft” brushes, i. e., *κ*_stiff_ = 20 *κ*_soft_. The brushes we are interested in modeling are very large (of the order of a few μm^17^). The DPD type of coarse graining integrates out all the internal degrees of freedom of a DPD particle, such as angle bending and torsional interactions^31^. A chain made of DPD particles connected by springs must be flexible then, because the persistence length of the repeating units grouped into the DPD particles is smaller than the particles themselves. Introducing an angular three—body interaction along the polymer backbone would have resulted in an unrealistically large Young’s modulus for the brushes; also, it must be kept in mind that as the degree of polymerization grows, the chains become fully flexible. Since DPD beads are larger than the persistence length, the internal rigidity of the molecules is absorbed at this coarse—grained level within the beads, and the collective stiffness or softness of the brush is adequately represented by the harmonic springs. The stiffness of the brushes can be controlled with the stiffness of the bonds joining the beads because there are many chains on the cell’s surface, which means the chains are in the ‘brush regime’: the average distance between the chains’ grafting sites is smaller than their radius of gyration. Therefore, polymer chains are forced to interact with one another and these chain—chain interactions prevent the brush layer from releasing compression by moving sideways when probed by the tip of the AFM. In effect, the brush becomes stiff, which is why solvent particles find it more difficult to penetrate it, as our simulations demonstrate. Hence, the collective behavior of the polymer chains with beads joined by stiff or soft springs is the key mechanism that determines the compressibility of the brush layer covering the cell.

To model an AFM, an opposite surface is introduced into the simulation box, parallel to the one where the brushes are attached, but one that is repulsive to the brushes and to the surrounding liquid particles (aqueous solvent, or buffer). Our model for the brush on normal cells is depicted in Fig. 1(a); the one corresponding to the cancerous cells is shown in Fig. 1(b). In addition to these brushes, monomeric solvent particles are included explicitly, which act like the buffer in AFM experiments on cells. Full details are provided in the Supplementary Information. The curve of the force applied by the AFM tip vs the distance between the tip and the cell surface obtained for the normal brush is compared with that obtained for a cancerous brush (both are of the liquid and soft types) in Fig. 1(c). The equivalent curves obtained from AFM measurements on healthy and cancerous human cervical epithelial cells^17^ are shown in Fig. 1(d). The trends of the curves predicted by the simulations agree with those found in the experiments namely, cancerous cells are softer than normal ones^3^,^4^,^5^,^6^,^9^,^14^. In both the computationally obtained (Fig. 1(c)) and experimental (Fig. 1(d)) force profiles, the force measured at a given compression distance is larger for the brushes on normal cells than for those on the cancerous cells. There appears a more rapid decay in the force profile of the brushes on cancerous cells than in the profile on normal ones, although such decay is more abrupt in the experimental profiles^17^. This occurs because the number of brushes of the smallest size (green brushes in Fig. 1(b)) per surface area on the cancerous cells is larger in the experiments than in the simulations. However, our calculations capture the qualitative difference between the mechanical response of brushes on normal and cancerous cells; additional evidence is provided by the Supplementary Figs S1(a) and S1(b), where it is shown that the force profile obtained from simulations of normal cells fits a single—brush model, while the one corresponding to the cancerous cells is best fit by a three—brush model, in full agreement with experiments^17^.

**Figure 2:**
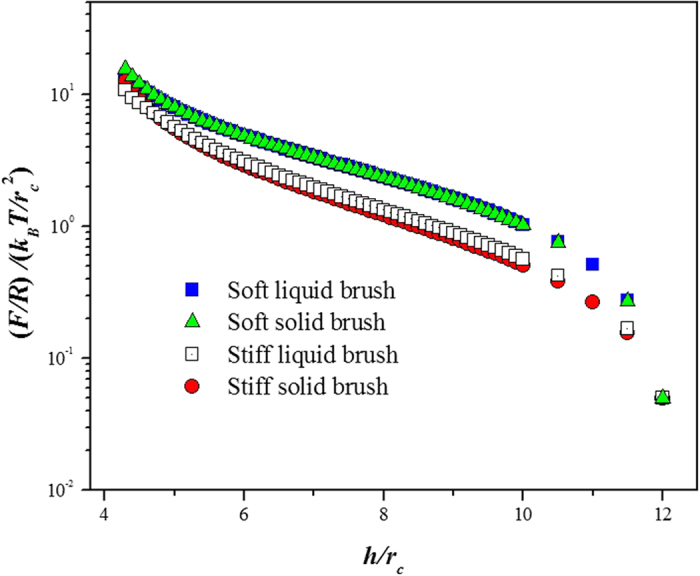
Force profiles for various types of brushes on cancerous cells. The *y*—axis represents the force (*F*) normalized by the radius of the AFM tip, *R*, (see Fig. 1(b)) that needs to be applied to obtain a deformation *h/r*_*c*_ of the cells’ brushes; *k*_*B*_ is Boltzmann’s constant, *T* the absolute temperature and *r*_*c*_ is the size of the DPD beads, *r*_*c*_ = 6.46 Å. Both types of *soft* brushes on cancerous cells (liquid and solid) exert a *larger* force on the AFM probe to be deformed the same distance *h/r*_*c*_ than their stiff brush counterparts. The number of beads that make up the brushes (*N*), as well as the number of brushes per unit area on the cell surface (Γ) are the same in all four cases shown in the figure, i. e., all brushes are made of *N*_1_ = 5, *N*_2_ = 30, *N*_3_ = 42 beads and Γ_1_ = 1.76 nm^−2^, Γ_2_ = 0.49 nm^−2^, Γ_3_ = 0.20 nm^−2^ brushes per unit area, respectively. The axes are shown in reduced units (see Methods and Supplementary Information).

In the rest of this work we focus on brushes on cancerous cells only, taking advantage of the fact that the simulations allow one to have control over the softness and mobility of the brushes on the cell’s surface. Figure 2 shows the force profiles obtained for all the types of brushes on cancerous cells we modeled. The data show that soft brushes require of a larger force to be compressed the same distance, *h*, than the stiff brushes. The liquid or solid nature of the brush is found to play a secondary role^32^. These results could be helpful for the understanding of how cancerous cells become softer as the disease progresses, how such softness promotes motility within the extracellular matrix, and can guide the search for mechanisms that increase brush stiffness to prevent metastasis^1^,^2^,^3^,^4^,^5^,^33^.

**Figure 3:**
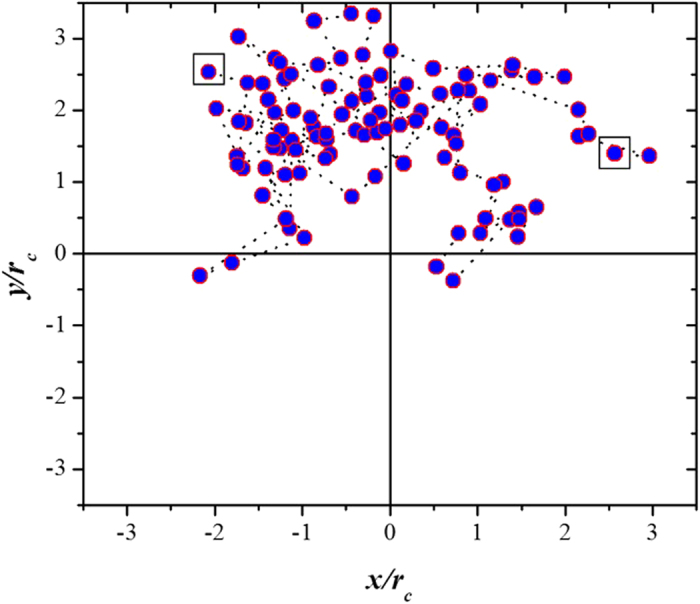
Meandering of a soft *liquid* brush “molecule” on the surface of a cancerous cell. Filled circles represent the positions on the *xy*—plane of the attached end of a randomly chosen liquid brush “molecule” on the surface of the cancerous cell, at intervals of 10^2^ Monte Carlo steps between one filled circle and the next. The squares indicate the positions of the “molecule” at the beginning (upper right quadrant), and at the end (upper left quadrant) of the meandering process, which was recorded over 10^4^ Monte Carlo steps. The compression distance between the AFM tip and the cell’s surfaces for this case is *h*/*r*_*c*_ = 12.0. The scales on both axes are expressed in reduced DPD units.

The motion of the chains that make up the so called liquid brushes on the surface of the cell can be considerable, as Fig. 3 shows, where the meandering of a given brush “molecule” on the surface of the cell is followed. However, such motion does not change the force profile with respect to the case when the brush molecules are fixed on the surface of the cell (solid brush) because the tip of the AFM covers the entire brush regardless of the type (liquid or solid), and the number of chains per unit area is the same for both types of brushes^34^. If the area of the AFM tip is smaller than that of the brushes being probed, a smaller force is expected for liquid brushes, since some of the brush molecules can relieve compression by escaping out of the region of deformation^21^.

**Figure 4:**
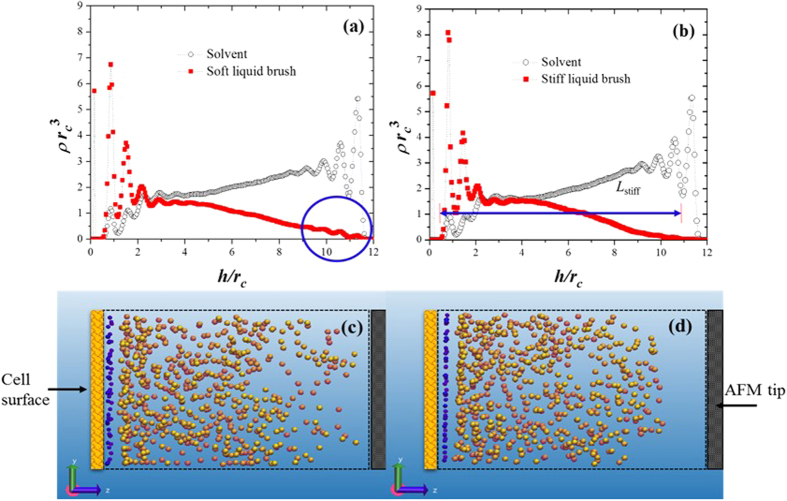
Bead number density profiles for two models of liquid brushes on cancerous cells. (**a**) Density profiles of the solvent (black empty circles) and the brush (red solid squares) modeled as soft chains. (**b**) Density profiles when the brushes are modeled as chains made up of beads connected with stiff springs. Notice how the stiff liquid brush, solid squares in (**b**), is almost unperturbed by the AFM tip, while the soft liquid brush (solid squares in (**a**)) does show some layering produced by the AFM tip, indicated by the blue circle. This occurs because the equilibrium brush lengths (*L*) are different: *L*_stiff_ < *L*_soft_. (**c**) Snapshot of the simulation of a soft brush on cancerous cells; the blue dots are the ends of the brush, which are allowed to move on the *xy*—plane, but not in the *z*—direction. (**d**) Snapshot of the stiff brush on cancerous cells; notice that the AFM does not touch the brushes. Only the beads that make up the brushes are shown in (**c**) and (**d**); the dotted rectangle represents the simulation box, which is of the same size in (**c**) and (**d**). The bonds joining the beads of the brushes and the solvent particles are omitted for clarity.

To determine why soft brushes are harder to compress than stiff ones, we show in Fig. 4(a,b) the density profiles of the beads that make up the soft and stiff brushes, respectively. The density profiles of the aqueous solvent particles are shown also. The analysis is focused only on liquid brushes, as appropriate for those on epithelial cells^33^. The reason why soft brushes require a stronger force than stiff brushes to produce the same deformation is that the equilibrium length of the soft brush, *L*_soft_, i.e. its length when is not yet compressed by the AFM tip, is larger than that of the stiff brush, *L*_stiff_, see snapshots in Fig. 4(c,d) and Supplementary Fig. S3; that explains why structuring appears in the part of the soft brush highlighted by the circle in Fig. 4(a). A large spring constant means that the monomers that make up the stiff brushes attract each other more strongly, leading to a shorter equilibrium length than that of their soft brush counterparts. A soft brush allows for an easier penetration of solvent molecules, as illustrated in Fig. 4(a) in the interval 2 ≤ *h*/*r*_*c*_ ≤ 5 and compared with the corresponding interval in Fig. 4(b), which translates into a larger osmotic pressure. Hence the force required to produce a given indentation on a soft brush is, paradoxically, larger than the one required to induce the same indentation on a stiff brush. This argument can be clearly seen from an analysis of the Alexander—de Gennes scaling law for the osmotic pressure, from which the force acting on the brush can be derived^35^:


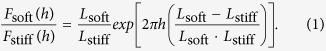


Equation (1) is valid for 0.1 < *h*/*L* < 0.9. Since the simulations show *L*_soft_ > *L*_stiff_, from equation (1) one finds that *F*_soft_ > *F*_stiff_ at a given value of *h*.

In conclusion, our simulations show that the lack of uniformity in thickness of the brushes on epithelial cancerous cells is responsible for their softness in comparison with normal cells. Biological brushes such as those on the surfaces of cells, as well as proteins on membranes, can move on the surface of the cell or membrane. However, we have shown for the first time that if the area of the AFM tip probes the entire surface on which the brushes are mobile, then the AFM tip records the same force for liquid and for solid brushes. This conclusion is important for those workers carrying out research with colloidal—sized AFM probes; for nanometer—sized probes a smaller force is expect for liquid brushes because some of the chains can relieve pressure by moving out of the area of compression. Our calculations show that larger forces are needed to produce indentation in soft brushes than in stiff ones on cancerous cells because the former allow for the penetration of more solvent particles, increasing the osmotic pressure. This stresses the need for the careful consideration of the buffer in AFM experiments with cells *in vitro*, since it can enhance or reduce the brush thickness, just as it occurs with grafted polymers under good or theta solvent conditions^18^. These conclusions are same whether the brushes are liquid, like microvilli on epithelial cells, or solid brushes, as in chemically adsorbed polymers on colloids (see Supplementary Fig. S4). Lastly, this work can be used as a guide to study how the stiffness of the brush correlates with the weakening of the actin filaments^36^ that make up the microvilli and explore the connection between brush stiffness and cancer stadia.

## Methods

The DPD model^23^,^24^ includes conservative, dissipative and random repulsive forces, whose strength is given by constants. The conservative force constant was chosen as equal for interactions between particles of the same type, while for solvent—brush interactions it was chosen as slightly more repulsive. The brushes were made up of linear chains of beads, freely joined with harmonic springs. For normal cell brushes the spring constant was *κ* =  100 (*k*_*B*_*T*/*r*_*c*_^2^). Stiff brushes on cancerous cells were modeled with spring constant *κ*=2000 (*k*_*B*_*T*/*r*_*c*_^2^); soft ones with *κ* = 100 (*k*_*B*_*T*/*r*_*c*_^2^). The area of the cells studied in all cases was *A* = 20.5 nm^2^; the radius of the AFM tip was chosen as *R* = 2.5 μm, and the distance range between it and the cell was 2.8 nm ≤ *h* ≤ 17.5 nm. The simulations were carried out using the Monte Carlo method, solved with the Metropolis algorithm, for a fluctuating number of solvent particles to keep the chemical potential fixed (grand canonical ensemble). Full details and additional analysis are presented in the Supplementary Information.

## Additional Information

**How to cite this article**: Gama Goicochea, A. and Alas Guardado S. J. Computer simulations of the mechanical response of brushes on the surface of cancerous epithelial cells. *Sci. Rep.* **5**, 13218; doi: 10.1038/srep13218 (2015).

## Supplementary Material

Supplementary Information
