# Supplementary material for: Computer simulations of the mechanical response of brushes on the surface of cancerous epithelial cells
Source: Sci Rep. 2015 Aug 28;5:13218. doi: 10.1038/srep13218 (PMC4551992; doi:10.1038/srep13218)
Supplement: Supplementary Information [file srep13218-s1.pdf]

Supplementary Information

Computer simulations of the mechanical response of brushes on the surface of cancerous epithelial cells

A. Gama Goicochea[1*] **and S. J. Alas Guardado**[2]

[1]Instituto de Física, Universidad Autónoma de San Luis Potosí

Álvaro Obregón 64, San Luis Potosí 78000, Mexico

[2]Departamento de Ciencias Naturales, Universidad Autónoma Metropolitana Unidad

Cuajimalpa, Av. Vasco de Quiroga 4871,

D. F. 05300, Mexico

SUPPLEMENTARY MODELS AND METHODS

The dissipative particle dynamics (DPD) model [S1] is made up of three forces that act on particles whose motion can be determined by solving Newton's second law of motion for the total force acting on each particle using finite time steps to calculate the momenta and positions of each particle at each time step. The force acting between any two particles *i* and *j* in DPD is given by a conservative force $(\boldsymbol{F}_{ij}^{C})$, a dissipative force $(\boldsymbol{F}_{ij}^{D})$, and a random one, $(\boldsymbol{F}_{ij}^{R})$. The total force acting on any given pair of particles, *i* and *j*, is the sum of these three forces:

$$\boldsymbol{F}_{ij} = \sum_{i\neq j}^{N} [\boldsymbol{F}_{ij}^{C} + \boldsymbol{F}_{ij}^{D} + \boldsymbol{F}_{ij}^{R}]. \qquad \text{(S1)}$$

* Corresponding author. Electronic mail: agama@alumni.stanford.edu

The conservative force is given by a soft, linearly decaying repulsive function:

$$\boldsymbol{F}_{\boldsymbol{ij}}^{\boldsymbol{C}} = \begin{cases} a_{ij}(1 - r_{ij})\hat{\boldsymbol{r}}_{\boldsymbol{ij}} & r_{ij} \le r_c \\ 0 & r_{ij} > r_c \end{cases}, \tag{S2}$$

where $\boldsymbol{r_{ij}} = \boldsymbol{r_i} - \boldsymbol{r_j}$, $r_{ij} = |\boldsymbol{r_{ij}}|$, $\hat{\boldsymbol{r}}_{\boldsymbol{ij}} = \boldsymbol{r_{ij}}/r_{ij}$, r_{ij} is the magnitude of the relative position between particles i and j, and a_{ij} is the intensity of the repulsion between a pair of particles. This interaction constant depends on the coarse – graining degree [S2], i. e., on the number of water molecules grouped into a DPD bead. In all our simulations we use a coarse – graining degree equal to three, which leads to $a_{ij} = 78.0(k_B T/r_c)$ for all types of particles, except for the interaction between solvent particles and the monomers that make up the polymeric brushes; in such case, $a_{ij} = 79.3(k_B T/r_c)$; k_B is the Boltzmann's constant and T the absolute temperature. The dissipative and the random forces are, respectively:

$$\boldsymbol{F}_{\boldsymbol{ij}}^{\boldsymbol{D}} = -\gamma\omega^D(r_{ij})[\hat{\boldsymbol{r}}_{\boldsymbol{ij}} \cdot \boldsymbol{v}_{\boldsymbol{ij}}]\hat{\boldsymbol{r}}_{\boldsymbol{ij}} \tag{S3}$$

$$\boldsymbol{F}_{\boldsymbol{ij}}^{\boldsymbol{R}} = \sigma\omega^R(r_{ij})\xi_{ij}\hat{\boldsymbol{r}}_{\boldsymbol{ij}}, \tag{S4}$$

where σ is the noise amplitude and γ is the friction coefficient and they are related as follows: $k_B T = \sigma^2/2\gamma$; $\boldsymbol{v}_{ij} = \boldsymbol{v}_i - \boldsymbol{v}_j$ is the relative velocity between the particles, and $\xi_{ij} = \xi_{ji}$ is a random number uniformly distributed between 0 and 1 with Gaussian distribution and unit variance. The weight functions ω^D and ω^R depend on distance and vanish for $r > r_c$, and are chosen for computational convenience to be (see [S1]):

$$\omega^D(r_{ij}) = [\omega^R(r_{ij})]^2 = max\left\{\left(1 - \frac{r_{ij}}{r_c}\right)^2, 0\right\}. \tag{S5}$$

All forces between particles i and j vanish further than a finite cutoff radius r_c, which represents the inherent length scale of the DPD model and it is regularly chosen as the

reduced unit of length, r_c = 1. The constants in equations (S3) and (S4) are chosen as σ = 3 and γ = 4.5, so that k_BT = 1 [S3], for all the cases reported in this work because those constants fix the thermostat, which should be the same for all types of brushes modeled here.

The DPD particles that make up the brushes and the buffer in this work are confined by a simulation box which is flanked by effective square surfaces placed at the ends of the box, perpendicularly to the z – axis, i.e. at z = 0 and $z = L_z$. The force model for those surfaces is a simple, linearly decaying force law in the z – direction. It is given by:

$$\boldsymbol{F}_{\boldsymbol{wall}}(z_i) = \begin{cases} a_w(1 - z_i/z_C)\hat{\boldsymbol{z}} & z_i \leq z_c \\ 0 & z_i > z_c \end{cases}. \tag{S6}$$

There are more sophisticated DPD wall models [S4], but for the purposes of the present work the model in equation (S6) is sufficient because it represents a soft wall, as a cell's surface is. It is important to emphasize that this model does allow for the deformation of cells' surfaces, as well as the brushes attached to them. The wall force in equation (S6) is used to model both the surface of the epithelial cervical cells where the brushes are attached, and the probe of the atomic force microscope (AFM), using however different values of the interaction strength of such force, a_w. To fix the ends of the brush molecules that are to be attached to the cell's surface we chose a_w = 60.0 (k_BT/r_c) while the interaction of the rest of the monomers in the brush with the cell's surface was chosen as a_w = 140.0 (k_BT/r_c). The interaction of the solvent particles with the surface of the cell was set at a_w = 140.0 (k_BT/r_c) also. As for the interaction of solvent and brush particles with the opposite surface, which is the tip of the AFM, they were all chosen as a_w = 140.0 (k_BT/r_c). To model "liquid" brushes [S5] we allowed the attached ends of the polymeric brushes to move freely on the surface of the cell (the xy – plane), while for the modeling of "solid" brushes, those ends were fixed at

random positions on the xy – plane. The brush chains were modeled as linear chains made up of identical beads which are joined by freely rotating harmonic springs [S6]:

$$\boldsymbol{F}_{spring}(\boldsymbol{r}_{ij}) = -\kappa_0(r_{ij} - r_0)\hat{\boldsymbol{r}}_{\boldsymbol{ij}}, \tag{S7}$$

where the spring constant, κ_0, was set at $\kappa_0 = 100.0\ (k_BT/r_c^2)$ for the so – called "soft" brushes, including those on normal cells; for the "stiff" brushes we chose $\kappa_0 = 2000.0\ (k_BT/r_c^2)$. The springs' position of equilibrium, r_0, was chosen in all cases as $r_0 = 0.7r_c$ [S7]. All beads that make up the polymer chains that model the brushes on normal and cancerous cells have the same size (r_c), which is larger than the persistence length of the brush "molecules". Therefore torsional angles and vibrations of angles between bonds are important only within beads, and the relatively long chains that represent the microvilli become fully flexible and can be modeled accurately with equation (S7) [S8-S10].

The central purpose of this work is the calculation of the force that the tip of the AFM exerts on the surface brushes of normal and cancerous cervical cells, which is obtained through the disjoining pressure, Π [S11]. It is defined as

$$\Pi(h) = P_N(h) - P_B, \tag{S8}$$

where $P_N(h)$ is the component of the pressure tensor that is perpendicular to the xy – plane, i. e., is the pressure exerted on the surfaces that confine the complex DPD fluid made up of polymer chains and solvent monomers. The bulk pressure, P_B, is obtained from the average of the diagonal of the pressure tensor of the isotropic system, namely the one without confining surfaces. The components of the pressure tensor are calculated using the virial theorem route [S12], which provides kinetic and "potential" contributions to the pressure

tensor. These components of the pressure tensor were calculated following the model of Irving and Kirkwood [S13], given as follows:

$$P_{xx} = \sum_i m_i \vec{v}_i \cdot \vec{v}_i + \sum_i \sum_{j>i} F_{ijx}^C x_{ij} \tag{S9}$$

where the first sum is the kinetic contribution. The second term is the product of the x – component of the conservative DPD force acting between particles i and j, and the x – component of the r_{ij} vector (see equation (S2)). The pressure tensor components P_{yy} and P_{zz} are obtained by replacing y and z by x in equation (S9), respectively. The normal pressure component in equation (S8) is $P_N = P_{zz}$, when the fluid is confined, while $P_B = {}^1/_3\left(P_{xx} + P_{yy} + P_{zz}\right)$, when it has periodic boundary conditions in all sides of the simulation box. To model the tip of the AFM we used a flat surface, see Figs. 4(c) and 4(d) in the manuscript, because we would like to model a colloidal AFM probe, of radius R = 2.5 μm, as in some experiments [S14]. Because of the difference in scales of the brushes' thickness and the AFM probe one can use the so – called Derjaguin approximation [S15], where the force (F) per curvature radius (R) of the AFM tip can be obtained from the pressure in equation (S8) between flat surfaces as follows:

$$\frac{F}{2\pi R} = -\int \Pi(h)\, dh. \tag{S10}$$

Equation (S10) is valid when the distance between the two flat surfaces (h) is smaller than the curvature radius R, which is a condition satisfied by our simulations. The compression of the brushes on normal and cancerous cells was modeled in the range 2.8 nm ≤ h ≤ 17.5 nm and we used the value R = 2.5 μm [S14], therefore the use of the Derjaguin approximation is fully adequate in this work. This is relevant for the scale of the experiments because these

simulations focus on the scale where the “grafting density” of the liquid and solid brushes is equal, given that the tip of the AFM probes an area where there are brushes of different length and the force collected by the AFM is necessarily an average over such area.

We use a hybrid DPD-Monte Carlo (MC) algorithm in the grand canonical ensemble (fixed chemical potential, volume and temperature) as is required to reach the equilibrium state of all systems modeled under confinement [S11]. The dynamics part of the algorithm proceeds as follows: the motion of the DPD particles under the influence of forces given by equations (S1) – (S7) is solved through the integration of Newton’s second law of motion [S12], using the velocity Verlet algorithm, adapted to the DPD interaction model [S16]. The dynamics is solved for ten time steps to generate a configuration, which is then compared with the initial configuration, after that the Metropolis algorithm is applied to choose between one of those two configurations [S17]. Once a configuration is chosen, the algorithm proceeds to try inserting and deleting aqueous solvent monomers a number of times, keeping the chemical potential of the solvent fixed. Full details of the DPD-MC method have been reported elsewhere [S11].

SYSTEMS STUDIED AND SIMULATION DETAILS

Dimensionless units are used throughout this work. Length is reduced with the cutoff radius, r_c which, for a coarse – graining degree equal to three water molecules per DPD particle is equal to r_c = 6.46 Å [S2]. The time step δt is reduced with $\delta t = (m r_C^2 / k_B T)^{1/2}$, where m is the mass of a DPD particle; using the mass of three water molecules in a DPD bead, δt = 6.3 ps at T=300 K. The reduced time step used in the dynamics part of the algorithm was in all cases δt/(6.3 ps) = 0.02; the energy was reduced with $k_B T$, with T = 300 K. The area of the

simulation box was fixed in all cases with $L_x/r_c = 7$, $L_y/r_c = 7$, while L_z/r_c was increased from 4.3 up to 27; periodic boundary conditions were used, except in the z-direction since this is the direction of the confinement. The chemical potential was fixed at μ=37.7k_BT, which gives a total average numerical density of $<\rho>$~$3/r_c^3$. The simulation results were obtained from averages after at least 600 blocks of 10^4 MC configurations each were carried out, with the first 100 blocks used to equilibrate the system and the rest were used for the production phase. In each given block made up of 10^4 MC configurations, the percentage of successful MC moves was around (35 ± 2) %.

The brushes on normal cells were modeled as linear chains made up of the same number of beads, N = 27, with one of their ends "grafted" to the cell surface but free to move on it (the so – called "liquid" type of brush). The spring constant joining those beads was chosen for all these chains as κ_0 = 100.0 (k_BT/r_c^2), which is called a "soft" brush. There were 16 chains attached to the surface of the normal cell, which yields a grafting density of Γ= 0.78 nm^{-2}. Brushes on cancerous cells were designed as non – uniform, following the experimental clues [S14]; they were made up of three different types of brushes. The shortest one was made of 36 chains with N_1 = 5 beads and grafting density Γ_1 = 1.76 nm^{-2}; the medium – sized brush consisted of 10 chains with N_2 = 30 beads and Γ_2 = 0.49 nm^{-2}, and the largest brush was made of 4 chains with N_3 = 42 beads with Γ_3 = 0.20 nm^{-2}. In contrast with the brushes on normal cells, the brushes on cancerous cells were allowed to have either fixed or moving ends on the surface (to model "solid" and "liquid" brushes, respectively), and the spring constant joining the beads along the chains was allowed to have two values: κ_0 = 100.0 (k_BT/r_c^2) for soft brushes, and κ_0 = 2000.0 (k_BT/r_c^2) for stiff ones.

SUPPLEMENTARY RESULTS AND DISCUSSION

In Fig. S1 we show the force that the AFM tip exerts on the brush that covers normal cells predicted by our simulations (circles in Fig. S1(a)) and those measured by Iyer and collaborators [S14] (circles in Fig. S1(b)). The trend predicted by our calculations is in very good agreement with that found in the experiments, although the scale of the distance between the AFM tip and the brush is orders of magnitude smaller. This is simply due to the shorter length of the brushes modeled in our simulations as compared to those in the experiments [S14]; for example, while the average length of the brushes on normal cells was found in experiments to be $L = 2360 \pm 970$ nm (see Supplementary Information of ref. [S14]), the corresponding equilibrium length of the brush on a normal cell was $L = 11.3$ nm in our simulations. It is the coarse – grained nature of our model for an AFM what enables us to capture the essential mechanical response of polymeric brushes.

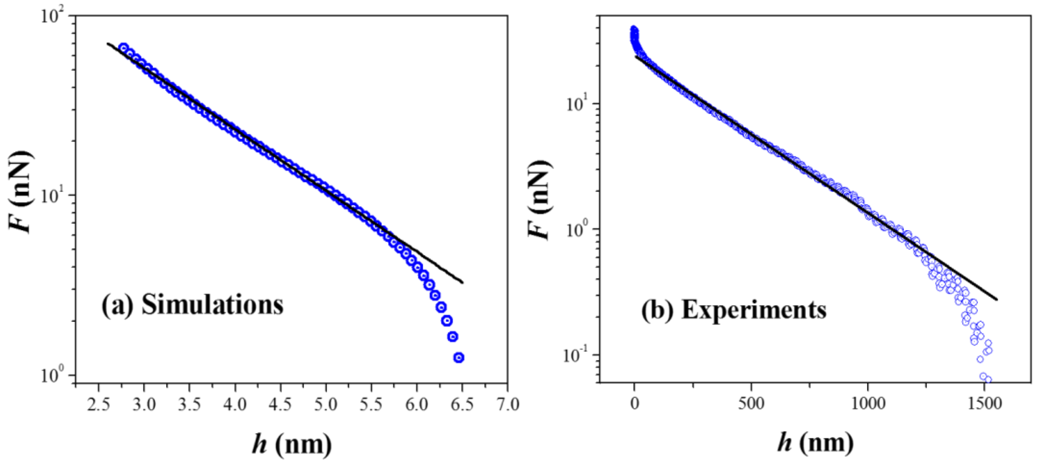

Figure S1. **Force profiles of brushes on normal cells.** (a) The force of the tip of an AFM acting of the brush covering normal epithelial cells, as predicted by our simulations (blue empty circles). (b) Force measured on normal human epithelial cervical cells (blue empty circles) with an AFM [S14]. The solid black line in both curves represents a best fit to the Alexander – de Gennes scaling equation for the force between polymer brushes [S15], see text for details of the fit.

Following Iyer and co – workers [S14] we have included in Fig. S1 the best fit to the Alexander – de Gennes scaling law for the osmotic pressure between a brush of uniform length and a solid surface [S18], which is given by:

$$F(h) = 50 k_B T R \Gamma^{\frac{3}{2}} L e^{-2\pi h/L} , \tag{S11}$$

where R is the radius of curvature of the AFM tip, Γ is the polymers' grafting density and L is the equilibrium length of the brush, namely its length when it is not compressed by the AFM tip. Equation (S11) is expected to be valid only when $0.1 < h/L < 0.9$. We have used equation (S11) to fit our data and the result is the black line in Fig. S1(a), which is given by the equation $F(h) = (540 \text{ nN}) e^{-2\pi h/L}$ with an effective equilibrium length L = 8 nm; in reduced DPD units this length is L/r_c= 80/6.46 ≈ 12.4, which is in good agreement with the equilibrium length obtained for the brushes in normal cells from simulations, as is indicated in Fig. S2. Our simulations have the advantage that the chains that make up the brushes do interact with one another, and do not assume that the brush density profile (Fig. S2) is a step function [S18]; none of these features are incorporated into the approximate scaling function in equation (S11) [S18]. Regarding our best fit of the data of Iyer and collaborators [S14] using equation (S11) (black line in Fig. S1(b)), we obtain $F(h) = (24.4 \text{ nN}) e^{-2\pi h/L}$, with equilibrium length L = 2175 nm, which is to be compared with the average experimental value, see the Supplementary Information in reference [S14]: 2360 ± 970 nm. Therefore, DPD simulations are a powerful tool to study the mechanical response of brushes on cells.

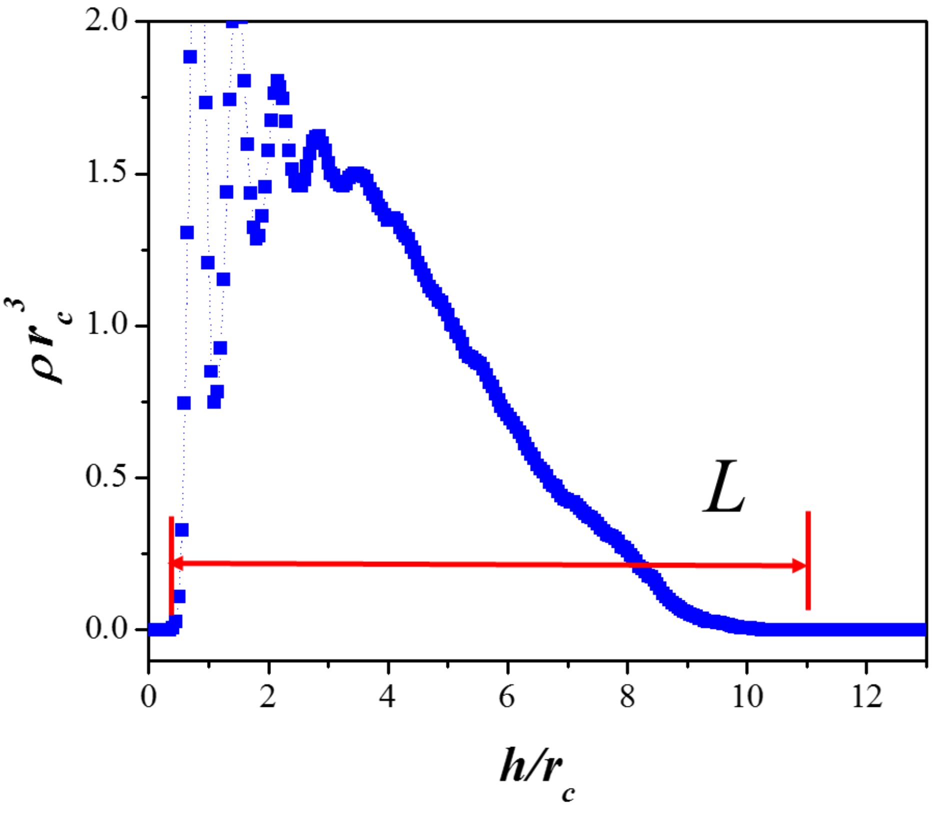

Figure S2. **Density profile of a brush on a normal epithelial cell predicted by simulation.** In this figure we see the number density of the beads that make up the chains of uniform length (solid squares), which is our model for the brush on normal cells, when the tip of the AFM is far from the brush, so that it can be considered to be in equilibrium. The equilibrium length of the brush (*L*) is indicated by the red line. Both axes are shown in reduced DPD units.

In Fig. S3 we show the density profiles of the soft and stiff liquid brushes in equilibrium, i. e., when the tip of the AFM is far from the brushes so that their length is not perturbed by the confining force of the tip. As we have argued in the main manuscript, in the stiff brush the beads that form the chains are closer to each other than in the soft brush, because the spring is "harder", hence the equilibrium length of the stiff brush (L_{stiff}) is shorter than that of the soft brush (L_{soft}). The soft brush then "swells" more than the stiff brush when molecules of the aqueous solvent penetrate it, leading to a larger force required to deform the soft brush

with the AFM than that required to deform the stiff brush. This argument follows also from equation (S11).

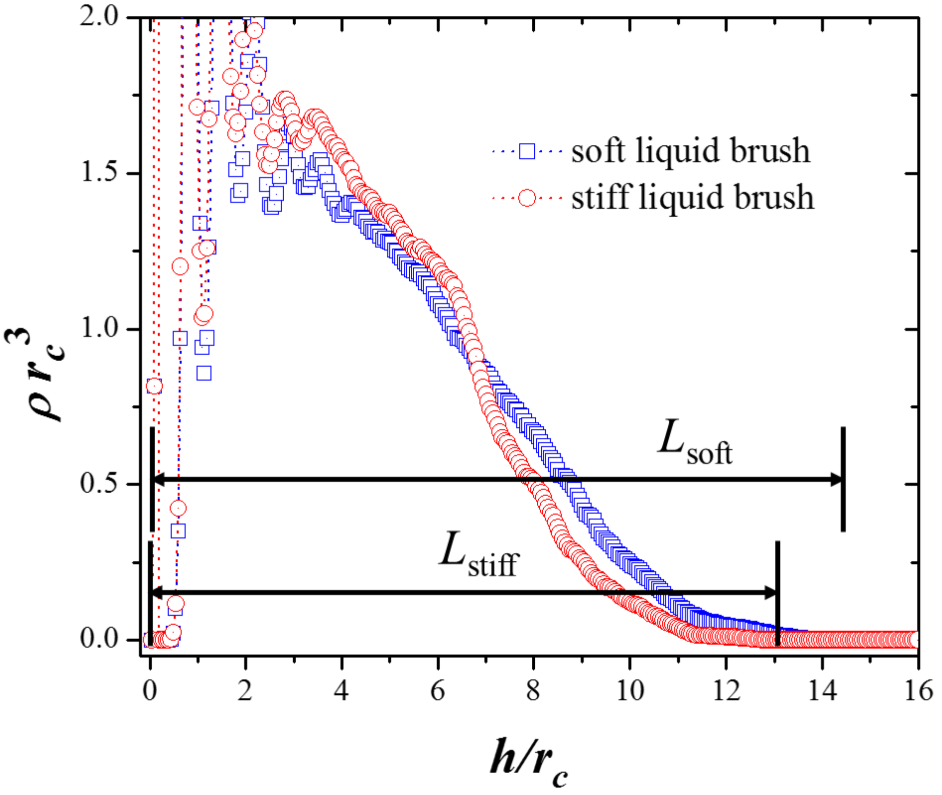

Figure S3. Comparison of brush density profiles on soft and stiff cancerous cells. Number density profiles of the beads that make up the brushes of non − uniform density, joined by stiff and soft harmonic springs, when the brushes are in equilibrium, that is, when the tip of the AFM is not in contact with any chain in the brushes. The ends of the chains of the brushes that are attached to the cell surface are allowed to move on the plane of the surface, i. e., the brushes are liquid. L_{soft} and L_{stiff} represent the equilibrium length of the soft and stiff liquid brushes, respectively.

In the experiments performed by Iyer and co – workers [S14] it was observed that cancerous cervical epithelial cells appeared to be covered by two rather dense brushes of different length, and a third brush of larger length than the other two, but of smaller "grafting density". Their force profiles also seemed to indicate the presence of such third brush, although the authors fit their data using only a two – brush Alexander – de Gennes type of model, simply

adding two terms of the type shown in equation (S11) with different parameters, see [S14]. In Fig. S4 we have followed the same approach to interpret the force profiles obtained from our simulations which, as we shall discuss, leads to important physical insights.

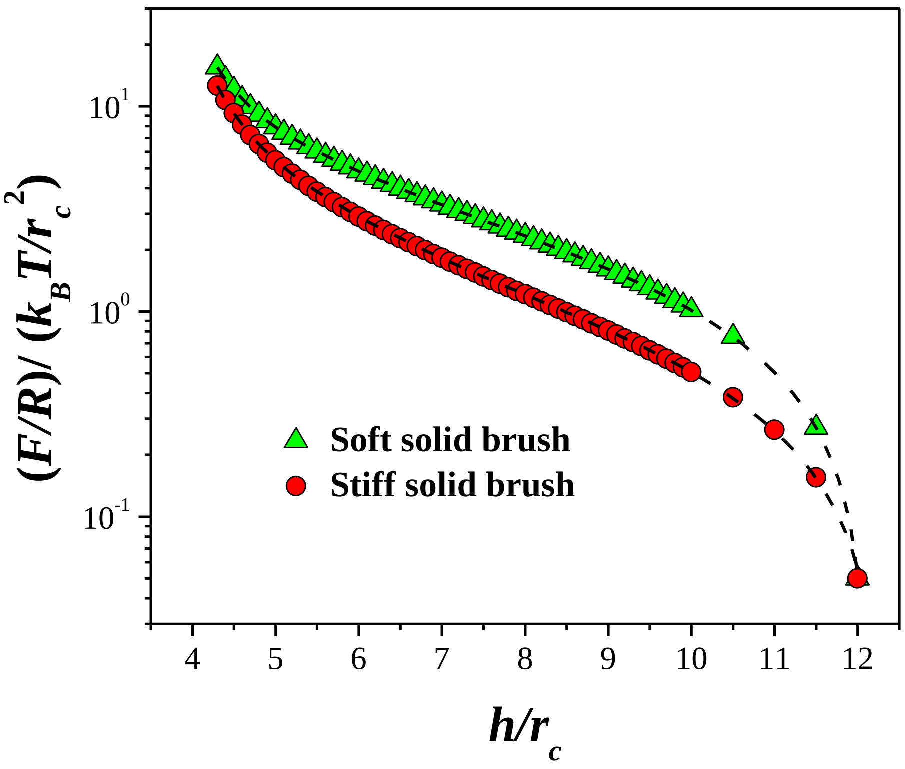

Figure S4. **Force profiles of brushes on soft and stiff solid brushes on model cancerous cells.** In this figure we show only the force per curvature radius of the AFM tip obtained for the so – called solid brushes on cancerous cells, i. e., those brush "molecules" that have fixed attaching sites on the surface of the cell. The case corresponding to the liquid brushes was discussed in the previous figure and in the main manuscript. Filled, green triangles are the data obtained for soft brushes, while filled (red) circles represent the force per radius on stiff brushes. The dashed lines are the best fits to a three – brush, Alexander – de Gennes type of model (see equation (S11)). See text for details.

In Fig. S4 we show the force profiles corresponding to the so – called solid type of brush only, since the characteristics of the liquid type have been discussed in detail in the previous paragraphs. The green triangles in Fig. S4 correspond to the force per curvature radius of the AFM tip when it compresses a soft, solid brush, while the red circles represent the equivalent profile, corresponding to the stiff solid brush. The dashed lines in Fig. S4 represent the best

fits to a three – brush, Alexander – de Gennes type of scaling for the force per curvature radius, namely:

$$\frac{F}{R}/\left(\frac{k_BT}{r_c^2}\right) = A_1e^{-(x-x_0)/L_1} + A_2e^{-(x-x_0)/L_2} + A_3e^{-(x-x_0)/L_3} + B. \quad \text{(S12)}$$

In equation (S12), A_i and L_i (with i = 1, 2, 3), x_0, and B are adjustable parameters. After performing regression analysis, we obtained the following values of these parameters for the **soft**, liquid brush (all are reported in reduced DPD units): A_1 = 4.91, L_1 = 0.293 ± 0.002; A_2 = 5.97, L_2 = 1.13 ± 0.02; A_3 = 9.01, L_3 = 9.8 ± 0.4, with x_0 = 4.28 and B = -4.1 ± 0.2. For the **stiff**, liquid brush the parameters are the following: A_1 = 5.72, L_1 = 1.06 ± 0.02; A_2 = 5.67, L_2 = 0.287 ± 0.003; A_3 = 3.91, L_3 = 7.0 ± 0.5, with x_0 = 4.23 and B = -1.2 ± 0.1. As can be observed in Fig. S4, the fit of equation (S12) to our predictions is excellent. Notice that the values obtained for the maximum compression of the brushes from this fit (x_0 = 4.28, and x_0 = 4.23 for soft and stiff brushes, respectively) are in very good agreement with the actual value from our simulations (x_0 = 4.3). More importantly, the fit yields an equilibrium length for the soft brush (L_{soft} = L_3 = 9.8 ± 0.4) that is **larger** than the equilibrium length of the stiff brush (L_{stiff} = L_3 = 7.0 ± 0.5), which confirms our conclusion that the force required to compress a soft brush is larger than that required to compress the same distance a stiff brush. We have shown here that this conclusion does not depend on the liquid or solid nature of the brushes, but only on the equilibrium length of the largest brushes the cover cancerous epithelial cells. Also, this work represents a successful coarse – grained model of an AFM cantilever interacting with a complex brush, which can be particularly useful when interpreting experiments on matter at the nanoscale, a task that still remains most challenging [S19].

SUPPLEMENTARY REFERENCES

[S1] Hoogerbrugge, P. J. & Koelman, J. M. V. A. Simulating microscopic hydrodynamic phenomena with dissipative particle dynamics. *Europhys. Lett.* **19**, 155 (1992).

[S2] Maiti, A. & McGrother, S. Bead-bead interaction parameters in dissipative particle dynamics: relation to bead-size, solubility parameter, and surface tension. *J. Chem. Phys*. **120**, 1594 (2004).

[S3] Español, P. & Warren, P. Statistical mechanics of dissipative particle dynamics. *Europhys. Lett.* **30**, 191 (1995).

[S4] Gama Goicochea, A. & Alarcón, F. Solvation force induced by exact, short range dissipative particle dynamics effective surfaces on a simple fluid and on polymer brushes. *J. Chem. Phys*. **134**, 014703 (2011).

[S5] Subramanian, G., Williams, D. R. M. & Pincus, P. A. Interactions between finite-sized particles and end grafted polymers. *Macromol*. **29**, 4045 (1996).

[S6] Murat, M. & Grest, G. S. Interaction between grafted polymeric brushes: a molecular – dynamics study. *Phys. Rev. Lett*. **63**, 1074 (1989).

[S7] Gama Goicochea, A., Romero-Bastida, M. & López-Rendón, R. Dependence of thermodynamic properties of model systems on some dissipative particle dynamics parameters. *Mol. Phys.* **105**, 2375 (2007).

[S8] Wang, T-Y., Fang, C.-M., Sheng, Y.-J., & Tsao, H-K. Osmotic pressure and virial coefficients of star and comb polymer solutions: dissipative particle dynamics. *J. Chem. Phys*. **130**, 124904 (2009).

[S9] Milchev, A. & Binder, K. Semiflexible polymers grafted to a solid planar substrate: changing the structure from polymer brush to “polymer bristle”. *J. Chem. Phys*. **136**, 194901 (2012).

[S10] Wijmans, C. M., Leermakers, F. A. M. & Fleer, G. J. Chain stiffness and bond correlations in polymer brushes. *J. Chem. Phys*. **101**, 8214 (1994).

[S11] Gama Goicochea, A. Adsorption and disjoining pressure isotherms of confined polymers using dissipative particle dynamics. *Langmuir* **23**, 11656 (2007).

[S12] Allen, M. P. & Tildesley, D. J. *Computer Simulation of Liquids*, Oxford University Press, New York (1989).

[S13] Irving, J. H., & Kirkwood, J. G. The Statistical Mechanical Theory of Transport Processes. IV. The Equations of Hydrodynamics. *J. Chem. Phys*. **18**, 817 (1950).

[S14] Iyer, S., Gaikwad, R. M., Subba-Rao, V., Woodworth, C. D. & Sokolov, I. Atomic force microscopy detects differences in the surface brush of normal and cancerous cells. *Nature Nanotech*. **4**, 389 (2009).

[S15] Israelachvili, J. N. in *Intermolecular and Surfaces Forces*, 2nd edn. (Academic Press, 1992).

[S16] Vattulainen, I., Karttunen, M., Besold, G. & Polson, J. M. Integration schemes for dissipative particle dynamics simulations: From softly interacting systems towards hybrid models *J. Chem. Phys.* **116**, 3967 (2002).

[S17] Frenkel, D. & Smit, B. *Understanding Molecular Simulation*, Academic Press, New York, 2002.

[S18] de Gennes, P. G. *Scaling Concepts in Polymer Physics*, Cornell University Press, New York, 1979.

[S19] O'Shea, S. J. & Welland, M. E. Atomic force microscopy at solid – liquid interfaces. *Langmuir* **14**, 4186 (1998).
